# High-dose etoposide could discriminate the benefit from autologous peripheral blood stem cell transplantation in the patients with refractory diffuse large B cell lymphoma

**DOI:** 10.1007/s00277-019-03605-1

**Published:** 2019-02-04

**Authors:** Yu Cai, Liping Wan, Juan Yang, Jun Zhu, Jieling Jiang, Su Li, Xianmin Song, Chun Wang

**Affiliations:** Department of Hematology, Shanghai General Hospital affiliated to Shanghai Jiaotong University, 100 Haining Road, Shanghai, 200080 People’s Republic of China

**Keywords:** Diffuse large B cell lymphoma, Refractory, High-dose etoposide, Autologous peripheral blood stem cell transplantation

## Abstract

To evaluate the strategy of using high-dose etoposide mobilization followed by autologous peripheral blood stem cell transplantation (APBSCT) in patients with diffuse large B cell lymphoma (DLBCL) refractory to rituximab-based chemotherapy. Forty patients with refractory DLBCL were treated with high-dose etoposide for stem cell mobilization. All patients were in progressive disease (PD) prior to mobilization and underwent high-dose chemotherapy followed by APBSCT. Successful PBSC mobilization was achieved in all patients. Twenty-three patients (57.5%) showed a clinical response to high-dose etoposide. After APBSCT, 17 patients (42.5%) achieved CR. The 2-year progression-free (PFS) and overall survival (OS) rate were higher in patients responding to high-dose etoposide (64.1% and 77.7%) compared to those without response (11.8% and 11.8%; *P* < 0.001 for both). The response to high-dose etoposide mobilization therapy was an independent prognostic factor for CR achievement, PFS and OS after APBSCT. High-dose etoposide mobilization chemotherapy followed by APBSCT could rescue a proportion of patients with refractory DLBCL who responded to etoposide mobilization regimen.

## Introduction

A significant proportion of patients with refractory diffuse large B cell lymphomas (DLBCL) fail to achieve CR or PR with conventional salvage therapy and never have a chance to proceed to transplantation, most often due to chemotherapy-resistant diseases [1–3]. According to NCCN guideline, the subgroup of patients who have progression or resistance to chemotherapy should be considered for a clinical trial because they are not candidates for autologous peripheral blood stem cell transplantation (APBSCT) [4].

APBSCT is one of the effective treatment modalities for refractory DLBCL, but only small proportion patients can achieve long-term remission [5, 6]. Most studies have shown that response to chemotherapy is one of the most important prognostic factors for these patients [7–9]. The results from the Grupo Español de Linfomas/Trasplante Autólogo de Médula Osea (GEL-TAMO) and Autologous Blood and Marrow Transplant Registry (ABMTR) data suggest that high-dose therapy (HDT)/APBSCT can be considered for the patients who do not achieve CR but still remain sensitive to chemotherapy [10–14]. Moreover, a proportion of the patients who are not sensitive to conventional salvage chemotherapy may still respond to high-dose chemotherapy and could benefit from the use of HDT/APBSCT [15]. It remains unclear how to select the patients with refractory lymphoma who will have benefit from HDT/APBSCT.

Etoposide (VP16), an epipodophyllotoxin, is one of the most frequently used mobilization chemotherapy agents for lymphoma without reciprocal resistance with other kinds of agents. Several groups have consistently used etoposide alone or in combination with other chemotherapy agents, which is very effective to mobilize peripheral blood stem cells (PBSCs) [16]. We hypothesized that high-dose etoposide for mobilization can not only reduce tumor burden in the patients but also would be helpful to identify the group of patients who might benefit from HDT/APBSCT.

In the present study, we evaluated the efficacy of high-dose etoposide in stem cell mobilization followed by APBSCT and identified a group of the patients with refractory lymphoma who benefitted from HDT/APBSCT after responding clinically to high-dose etoposide mobilization chemotherapy.

## Patients and methods

### Patients

**Fig. 1:**
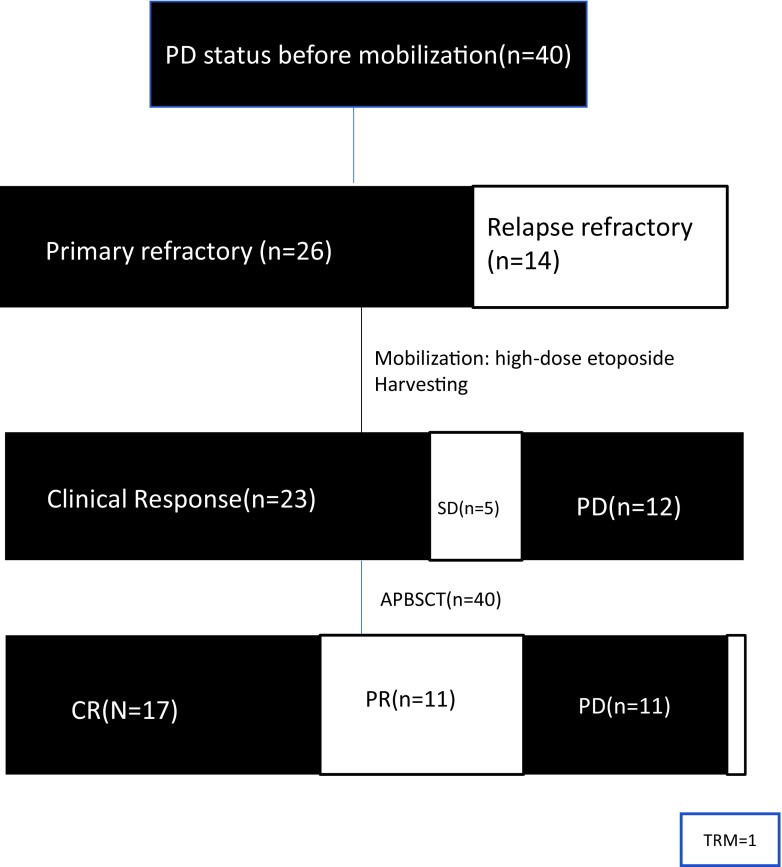
Consort diagram of patient distribution

We recruited 40 patients with histologically documented DLBCL at the Shanghai General Hospital from November 2005 to December 2016. A total of 28 male and 12 female patients with a median age of 39 years (range 16–61) were enrolled in this study. All patients had a diagnostic pathology. Lymphoma classification was performed according to the 2008 WHO myeloid and lymphocytic tumor diagnostic criteria. The criteria for relapsed (after CR) and refractory lymphoma were based on 2007 response criteria for non-Hodgkin lymphoma [17]. The primary refractory disease was defined as the cases that did not show any response to the first-line treatment. The relapsed refractory disease was defined as the cases without any response to salvage chemotherapy after relapse. The disease status was evaluated every 3 months after transplant. The study was approved by the Ethics Committee of Shanghai General Hospital (Clinical trial number 2015KY143) and conducted in accordance with the Declaration of Helsinki. We obtained a written informed consent from all recruited patients (or their legal guardians). This trial was registered at www.clinicaltrials.gov (Clinical trial number: NCT03130582). All patients were followed up to the end of December 2016 (Fig. 1).

Patient characteristics are summarized in Table 1. Thirty-four out of 40 (85.0%) patients had received at least three different kinds of rituximab-based chemotherapy regimens, including RCHOP (rituximab, cyclophosphamide, doxorubicin, vincristine, and prednisone), RICE (rituximab, ifosfamide, etoposide, cisplatin), RESHAP (rituximab, etoposide, methylprednisolone, high-dose cytarabine/cisplatin), and RDHAP (rituximab, cytarabine, dexamethasone, cisplatin). At least 6 cycles of prior chemotherapy were administered. At the time of enrollment, all patients had a PD status and above normal in lactic dehydrogenase level prior to mobilization (40/40, 100%).

**Table 1:** Patient characteristics

Characteristics	Number of patients (%)
Total	40
median Age, y (range)	39 (16–61)
≥ 45 years	29 (72.5%)
< 45 years	11 (27.5%)
Gender	
Male	28 (70.0%)
Female	12 (30.0%)
Histopathological classification	
Diffuse large B cell lymphoma	40 (100%)
Tumor bulk	
≥ 10 cm	7 (17.5%)
Number of prior chemotherapy regimens	
2	6 (15.0%)
3	12 (30.3%)
4 or more	22 (55.0%)
Number of prior chemotherapy cycles	
6	3 (7.5%)
8	5 (12.5%)
9 or more	32 (80.0%)
Previous radiation therapy	
Yes	4 (10.0%)
No	36 (90.0%)
B symptoms	25 (62.5%)
Extranodal disease	37 (92.5%)
Conditioning regiments	
BEAM	15 (37.5%)
CBV	21 (52.5%)
LACE	4 (10.0%)
Refractory status	
Primary refractory	26 (65.0%)
Relapse refractory	14 (35.0%)
ECOG	
0–2	17 (42.5%)
3–4	23 (57.5%)
Previous rituximab therapy	
Yes	37 (92.5%)
No	3 (7.5%)

### PBSC mobilization and harvesting

All patients received high-dose etoposide 20–25 mg/kg/day [18, 19] intravenously for 2 consecutive days followed by granulocyte colony-stimulating factor (GCSF, filgristim) 7 μg/kg/day subcutaneously at 48 h after etoposide infusion was completed in hospital.

Leukapheresis was performed with blood cell separator (Cobe Spectra, Terumo BCT, USA). The harvested cells reached at least 2 × 10^6^/kg of CD34^+^ cells with one to two leukapheresis procedures. The final product was kept frozen at − 80 °C.

### Autologous peripheral blood stem cell transplantation

Fifteen patients were treated with BEAM regimen, consisting of BCNU (300 mg/m^2^ on day 7), etoposide (150–200 mg/m^2^ on days 6, 5, 4, and 3), cytosine arabinoside (200–400 mg/m^2^ on days 6, 5, 4, and 3), and melphalan (140 mg/m^2^ on day 2). Twenty-one patients were treated with CBV regimen consisted of BCNU (300 mg/m^2^ on day 6), cyclophosphamide (1500–1800 mg/m^2^ on days 5, 4, 3, and 2), and etoposide (400–700 mg/m^2^ on days 5, 4, 3, and 2). Four patients were treated with LACE regimen consisting of BCNU (100 mg/m^2^ on day 8, 7, 6), cyclophosphamide (1200–1800 mg/m^2^ on days 8, 7, 6, and 5), Ara-C (1000 mg/m^2^ on days 4, 3, 2), and etoposide (400–700 mg/m^2^ on days 5, 4, 3, and 2). The preserved hematopoietic cells were re-infused on day 0 after they were thawed in a 42 °C water bath.

### End points and statistical methods

The response to chemotherapy and APBSCT was determined according to the 2007 response criteria for non-Hodgkin lymphoma [17]. Clinical response to high-dose etoposide was defined as the size of nodal masses decreasing by at least 20% [20], while not meeting PR criteria for the mobilization.

The primary end point used in our study was progression-free survival (PFS). The secondary end point was overall survival (OS). PFS was calculated as the date of APBSCT to the progression of lymphoma, death due to any cause if no progression, or last follow-up. OS was calculated as the date after APBSCT until death or the last follow up. Disease response was assessed according to the 2007 response criteria for non-Hodgkin lymphoma after day 30 post-APBSCT.

The OS and PFS were measured from the date of transplants, and they were estimated according to the Kaplan-Meier method. The log rank test was used to assess the difference in survival between each of the risk score values. Univariate and multivariate analyses for OS and PFS were performed using Cox proportional hazard regressions model. Logistics regression was performed for response including CR in the univariate and multivariate analysis. Variables significant at *P* < 0.1 were considered for multivariate analyses. *P* < 0.05 was considered statistically significant throughout the analysis. The statistical analyses were performed using SPSS19.0 statistical software and R 3.2.3 (R Development Core Team, Vienna, Austria) software package.

## Results

### Patient’s characteristics

Patient characteristics were summarized in Table 1. Twenty-six (65.0%) patients were primary refractory; others were relapsed refractory. All patients had an Ann Arbor stage of III–IV. Six patients underwent 36–40-Gy local radiotherapy before mobilization. No patients had bone marrow involvement before mobilization. Two patients who had no response to etoposide had received with 2–4 additional cycles of chemotherapy after mobilization due to PD. All patients were followed up for a median of 28.9 months (range, 1–121) after APBSCT.

### PBSC mobilization and APBSCT

Successful PBSC mobilization was achieved in all patients. The median number of CD34^+^ cells infused was 7.06 × 10^6^/kg (range, 2.82–41 × 10^6^/kg). The median nadir white blood cell count was 0.5 × 10^9^/L (range, 0.07–2.0 × 10^9^/L) after high-dose etoposide. Twenty-nine of 40 patients (72.5%) developed infection without sepsis and were successively treated with antibiotics. G-CSF was given for 7–20 days (median 13 days) to mobilize HSCs/HPCs. The median nadir platelet count was 34 × 10^9^/L (range, 3–122 × 10^9^/L) after high-dose etoposide.

### Clinical response to high-dose etoposide

After HSC/HPC mobilization and harvesting, no patients achieved PR or CR with high-dose etoposide. However, 23 of 40 patients (57.5%) showed a clinical response.

### Response to APBSCT

All but one patient had successful hematologic engraftment after APBSCT. The 52 years-old female patient (2.5%) died of failure to bone marrow reconstitution, which might be related with five different prior chemotherapy regimens with alkylating agents and poor perform status (ECOG 3) before mobilization, although the CD34^+^ cells infused to her was 2.82 × 10^6^/kg. The median time for neutrophil engraftment was 13 days (range, 11–21 days), whereas platelet counts above 20 × 10^9^/L were observed at a median time of 16 days (range, 11–25 days).

The median time from collection to re-infusion for APBSCT was 27 days (range 21–114). After APBSCT, 17 of 40 patients (42.5%) achieved CR and 11 (27.5%) patients attained PR, while 11 patients (27.5%) experience disease progression at 28 days (range 21–60).

### Survival

**Fig. 2:**
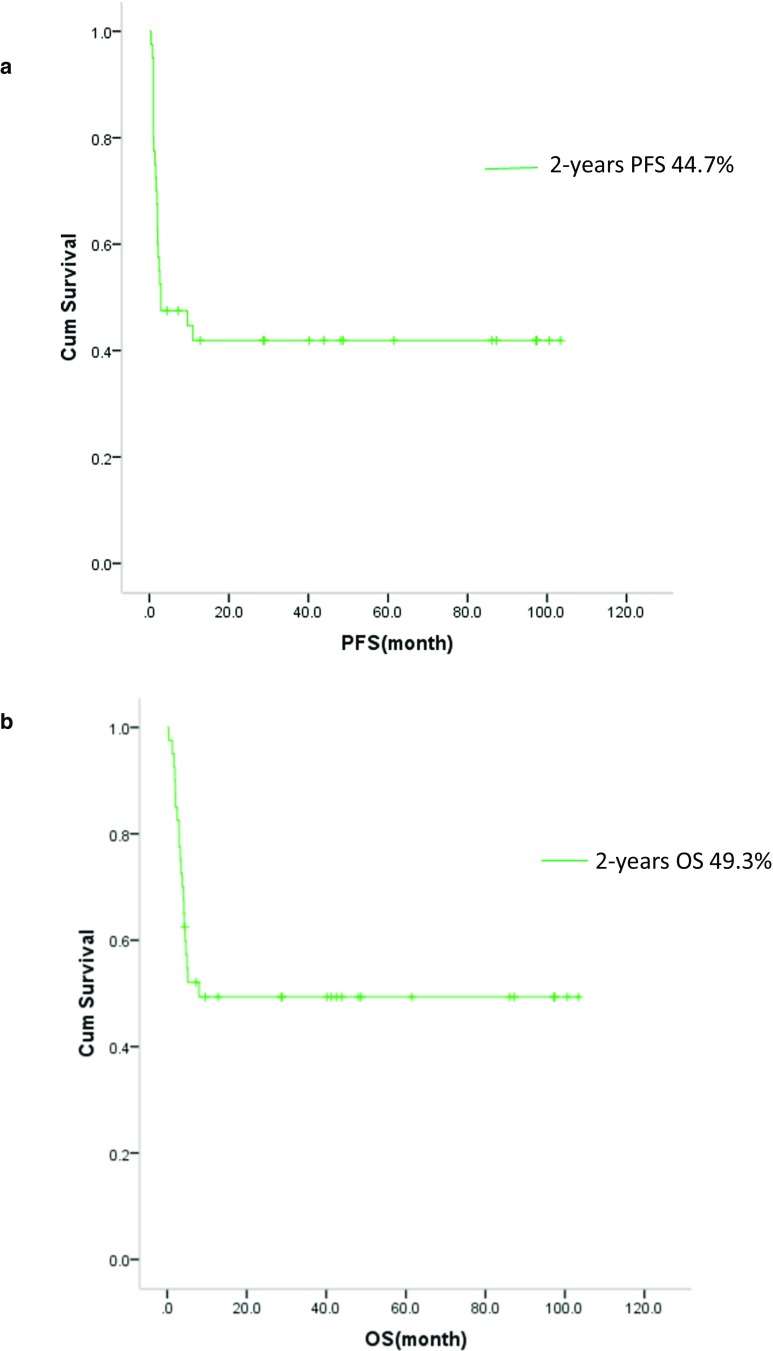
**a** Kaplan–Meier product estimate of the cumulative probability of progression-free survival (PFS) in patients (*n* = 40). **b** Kaplan–Meier product estimate of the cumulative probability of overall survival (OS) in patients (*n* = 40)

**Fig. 3:**
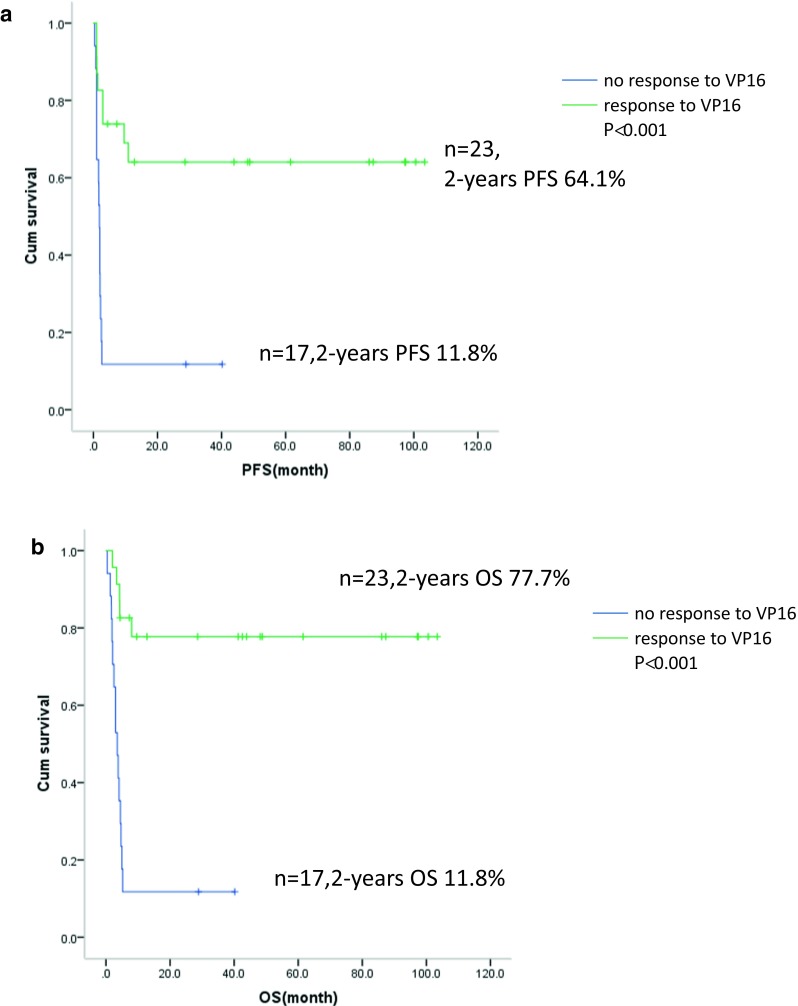
**a** Kaplan–Meier product estimate of the cumulative probability of progression-free survival (PFS) in patients who had response (*n* = 23) or no response to etoposide (*n* = 17). **b** Kaplan–Meier product estimate of the cumulative probability of overall survival (OS) in patients who had response (*n* = 23) or no response to etoposide (*n* = 17)

Clinical outcomes of patients in the study are illustrated in Fig. 1. The estimated 2-year PFS rate was 44.7%, 95%CI (29.2–60.2%) (Fig. 2a). The estimated 2-year OS rate was 49.3% (33.6–65.0%) (Fig. 2b). The 2-year PFS rate was significant higher in patients who had clinical response to high-dose etoposide (64.1% (44.1–84.1%) vs 11.8% (0.00–27.1%), *P* < 0.001)(Fig. 3a). Additionally, the 2-year OS rate was higher in patients who had a clinical response to high-dose etoposide (at 77.7% (95% CI: 60.4–94.9%)) than in those who did not have a clinical response (at 11.8% (0.0–27.1%), *P* < 0.001) (Fig. 3b).

The median time to progress after APBSCT was 6.4 (range 2.9–15.3) months. Two of 17 patients (11.8%) achieved CR after APBSCT had relapsed within 16 months of transplants. Eventually, 20 patients died, of whom one patient died early due to graft failure, and two patients died due to pulmonary infection at 4 and 13 months. The remaining patients died of disease progressive (*n* = 17).

### Univariate analysis for CR, PFS, and OS after APBSCT

Univariate analyses for CR, PFS, and OS after APBSCT were shown in Table 2. Using logistic regression model, three clinical factors were found to be associated with CR achievement after APBSCT: (1) clinical response to high-dose etoposide (odds ratio (OR) 6.623 (2.725–16.129), *P* < 0.001) and (2) conditioning regimens with BEAM (OR, 2.873 (1.706–6.802), *P* = 0.036) were demonstrated to have a significant impact toward a higher CR achievement after APBSCT. However, (3) primary refractoriness over relapse refractory disease was found to be an adverse prognostic factor with lower CR achievement (OR 0.071 (0.007–0.752), *P* = 0.048).

**Table 2:** Univariate analysis of factors associated with CR rate, PFS, and OS after APBSCT

Covariates	CR	*P*	PFS	*P*	OS	*P*
	OR (95% CI)		HR (95% CI)		HR (95% CI)	
Age (> 45 years)	1.382 (0.982–1.740)	0.752	0.760 (0.552–1.035)	0.598	0.905 (0.707–1.218	0.905
B symptoms (yes vs no)	1.009 (0.523–1.517)	0.985	0.831 (0.671–1.281)	0.536	1.209 (0.617–1.822)	0.271
Extranodal disease (yes vs no)	0873 (0.749–1.293)	0.164	1.304 (0.870–1.963)	0.585	1.069 (0.701–1.653)	0.648
ECOG (≥ 2 vs < 2)	1.727 (0.543–1.835)	0.051	**2.078 (1.062–4.066)**	**0.001**	**2.130 (1.095–4.146)**	**0.000**
Primary refractory (primary vs relapse)	**0.071 (0.007–0.752)**	**0.048**	0.799 (0.587–1.077)	0.874	0.968 (0.774–1.282)	0.912
Tumor bulk (≥ 10 cm vs < 10 cm)	1.234 (0.846–1.799)	0.093	0.876 (0.599–1.282)	0.588	0.975 (0.668–1.423)	0.537
Previous rituximab (yes vs no)	1.024 (0.682–1.540)	0.923	1.194 (0.830–1.742)	0.617	1.126 (0.736–1.679)	0.520
Clinical response to high-dose etoposide (yes vs no)	**6.623 (2.725–16.129)**	**0.000**	**0.139 (0.049–0.395)**	**0.000**	**0.124 (0.044–0.349)**	**0.000**
Conditioning regiments (BEAM vs CBV and LACE)	**2.873 (1.706–6.802)**	**0.036**	0.824 (0.672–1.006)	0.190	1.046 (0.014–5.547)	0.239

OR, odds ratio; HR, hazard ratio; 95% CI, 95% confidence interval; statistically significant differences shown in bold; CR, complete remission; OS, overall survival; PFS, disease-free survival

In Cox proportional hazard regression model for PFS, two clinical factors were found to be independent predictors: (1) clinical response to high-dose etoposide (HR, 0.139 (0.049–0.395), *P* < 0.001) was associated with longer PFS and (2) poor performance status with ECOG 2 or above was associated with shorter PFS (HR, 2.078 (1.062–4.066), *P* = 0.001). With respect to OS, (1) clinical response to high-dose etoposide (HR, 0.124 (0.044–0.349), *P* < 0.001) was associated with longer OS, and (2) poor performance status with ECOG 2 or above was associated with shorter OS (HR, 2.130 (1.095–4.146), *P* < 0.001).

### Multivariate analyses for CR, PFS, and OS after APBSCT

In order to confirm that those risk factors identified in the univariate analysis were independent factors, we performed multivariate analyses. According to the results of the univariate analysis, three covariates were selected for multivariate analysis, including ECOG, conditioning regiments and clinical response to high-dose etoposide (Table 3). The multivariate analyses of factors associated with CR achievement after APBSCT, PFS, and OS are presented in Table 3. By multivariate analysis, the clinical response to high-dose etoposide was demonstrated to have a significant impact or a marked trend toward a higher CR achievement (OR, 2.331 (1.481–3.891), *P* < 0.001), longer PFS (HR, 0.210 (0.069–0.643), *P* = 0.006), and OS (HR, 0.183 (0.062–0.543), *P* = 0.002). Poor performance status of ECOG 2 or above was also associated with shorter PFS (HR, 2.662 (1.123–6.039), *P* = 0.030) and shorter OS (HR, 3.832 (1.297–11.324), *P* = 0.024).

**Table 3:** Multivariate analysis of factors associated with CR rate, PFS, and OS after APBSCT

Covariates	CR	*P*	PFS	*P*	OS	*P*
	OR (95% CI)		HR (95% CI)		HR (95% CI)	
ECOG (≥ 2 vs < 2)	–	–	2.662 (1.123–6.309)	**0.030**	3.832 (1.297–11.324)	**0.024**
Clinical response to high-dose etoposide (yes vs no)	**2.331 (1.481–3.891)**	**0.014**	**0.210 (0.069–0.643)**	**0.006**	**0.183 (0.062–0.543)**	**0.002**
Conditioning regiments (BEAM vs CVB and LACE)	2.127 (0.612–4.042)	0.052	–	–	–	–

OR, odds ratio; HR, hazard ratio; 95% CI, 95% confidence interval; statistically significant differences shown in bold; CR, complete remission; OS, overall survival; PFS, disease-free survival

## Discussion

Here, we report the outcome of refractory DBLCL after high-dose etoposide mobilization chemotherapy followed by APBSCT. A proportion of patients (42.5%) was rescued with APBSCT, despite being refractory to chemotherapy for DLBCL. The response to high-dose etoposide was predictive for the CR achievement and prognostic for PFS, OS after APBSCT.

There are many studies in the literature, which demonstrate that the quality of response to salvage chemotherapy remains one of the strongest predictors for long-term survival in patients with refractory DBLCL undergoing APBSCT. Several studies showed that chemotherapy-resistance or suboptimal response to pretransplant salvage chemotherapy represents the major adverse prognostic factors affecting PFS [18–23]. Further, it has infrequently reported that the patients who were chemotherapy-resistant had successfully achieved long-term disease control after APBSCT. Vose JM et al. data showed that the patients who underwent APBSCT when the disease was resistant to the initial induction therapy had less than 20% probability of durable PFS [24]. Many patients in PD had no chance to undergo APBSCT. However, some of them who had no response to standard dose regimens may respond to the high-dose chemotherapy.

In the present study, clinical response to high-dose etoposide mobilization regimen was noted in 57.5% of patients, although they showed resistance to salvage regimens, such as RICE and R-DHAP. The 2-year PFS and OS rate was observed as 64.1 and 77.7% in the patients who had responded to the high-dose etoposide mobilization regimen, despite their lack of response to the previous lines of chemotherapy, which were mainly rituximab-based regimens. However, the PFS/OS rate was very low at 11.8% in the patients who did not show any response to high-dose etoposide, which suggests that the biologic nature of their lymphoma was really aggressive and had significantly highly chemotherapy-resistant. High-dose etoposide mobilization regimen could not only be used for stem cell mobilization but also to reduce tumor burden prior to APBSCT in PD patients. Additionally, it could help to identify a group of selective patients who can be rescued by the use of HDT followed by APBSCT, in the case of patients who have shown some chemotherapy-sensitivity with the high-dose etoposide regimen. The patients with truly refractory DLBCL may be defined as those with no clinical response to high-dose chemotherapy like etoposide. Alternately, patients with chemotherapy sensitivity may be defined as those with any clinical response to high-dose chemotherapy. These patients may benefit from APBSCT.

In the present study, we defined clinical response to high-dose etoposide according the criteria adopted by Dunleavy et al. who used a 20% reduction in the mass to assess the effect of the treatment [20]. Clinical response to high-dose etoposide was defined as the size of nodal masses decreasing by at least or more than 20% prior to mobilization. It was difficult to decrease nodal masses size in refractory lymphoma by more than 25% using single agents. In refractory patients of lymphoma, the use of single agents, such as etoposide, cisplatin, mitoxantrone lenalidomide [25], and paclitaxel [26], resulted in response rates from 20 to 40%; however, responses to monotherapy were generally not long lasting. The diseases were still sensitive to chemotherapy if the nodal masses decreased at least 20% or more than 20% after treatment with high-dose etoposide, indicating that subsequent greater intensity regimens followed by APBSCT were effective. In multivariate analysis, clinical response to high-dose etoposide was demonstrated to have a significant impact or a marked trend toward a higher CR achievement and longer PFS and OS.

Although high-dose cyclophosphamide followed by GCSF was the most commonly used chemotherapy/cytokine mobilization regimens [19, 27–29], cyclophosphamide is one of the agents in many conventional chemotherapy regimens for lymphoma. However, PD patients might be resistant to cyclophosphamide. Compared with cyclophosphamide, etoposide was less commonly used as a component of conventional first-line therapy. Therefore, etoposide might lack cross-resistance with cyclophosphamide. In addition, high-dose etoposide served as a test for chemotherapy sensitivity and to facilitate the harvest of stem cells. In the present study, the results showed that high-dose etoposide followed by G-CSF was an effective and useful method of mobilization in patients with refractory and relapsed lymphomas.

The type of transplantation offered to patients, based on patient selection and disease-related factors, can achieve long-term survival, highlighting the importance of further improvement in disease control and reducing procedure-related mortality. One potential clinical option for those patients that demonstrate to be refractory to high-dose etoposide is the possibility to receive allogeneic transplantation (allo-SCT) or a tandem transplantation procedure autologous stem cell transplantation as debulking therapy followed by allogeneic transplantation. Crocchiolo [29] et al. data showed that after allogeneic stem cell transplantation, the 3-year OS, PFS, non-relapse mortality, and relapse/progression were 68 (95% CI 59–77), 61 (95% CI 52–70), 17 (95% CI 10–25), and 22% (95% CI 14–30). Nishitha M [30] et al. data showed that 5-year OS rates between APBSCT and allo-SCT were 59% (95% CI, 51–66%) vs 52% (95% CI, 40–63%), *P* = 0.5, respectively. Five-year PFS were 51% (95% CI, 42–58%) vs 49% (95% CI, 37–60%). It is indicated that allo-HCT is not superior to auto-HCT as the first line in lymphoma patients. But allo-HCT may be an option of salvage therapy in the patients who do not benefit from auto-HCT [31]. Another promising option may be CAR-T for refactory lymphoma patients [32, 33].

In conclusion, stem cell mobilization with high-dose etoposide and G-CSF followed by APBSCT was beneficial in the treatment of refractory DLBCL. Utilizing high-dose etoposide, we could select some chemotherapy-sensitive patients with refractory DLBCL who would benefit from additional treatment. Patients’ clinical response to high-dose etoposide was correlated to a better outcome of APBSCT. Future prospective clinical trials should aim to consider the benefit in these highly refractory DLBCL patients with cell of origin, double/triple hit expression by high-dose etoposide mobilization regimen followed APBSCT.
